# Meta-Analysis of the Effect of Exercise on Neuropathic Pain Induced by Peripheral Nerve Injury in Rat Models

**DOI:** 10.3389/fneur.2019.00636

**Published:** 2019-06-14

**Authors:** Jia-bao Guo, Bing-lin Chen, Ying Wang, Yi Zhu, Ge Song, Zheng Yang, Yi-li Zheng, Xue-qiang Wang, Pei-jie Chen

**Affiliations:** ^1^Department of Sport Rehabilitation, Shanghai University of Sport, Shanghai, China; ^2^The Second Clinical Medical School, Xuzhou Medical University, Xuzhou, China; ^3^The Fifth Affiliated Hospital of Zhengzhou University, Zhengzhou, China

**Keywords:** running, swimming, NP, peripheral nerve injury, animal model, meta-analysis

## Abstract

**Background:** There is accumulating evidence showing that exercise therapy may play an active role in peripheral neuropathic pain (NP). However, there have been no meta-analysis to investigate the effects of exercise on NP induced by peripheral nerve injury in rat models.

**Methods:** PubMed, EMBASE, and Web of Science were searched from inception to January 2019. A random-effect model was implemented to provide effect estimates for pain-related behavioral test outcome. Mean differences (MDs) with 95% confidence intervals (CIs) were calculated.

**Results:** Fourteen studies were included. For the mechanical withdrawal threshold, rats in the exercised group exhibited significantly higher thresholds than those in the control group, with a MD of 0.91 (95% CI 0.11–1.71), 3.11 (95% CI 1.56–4.66), 3.48 (95% CI 2.70–4.26), 4.16 (95% CI 2.53–5.79), and 5.58 (95% CI 3.44–7.73) at 1, 2, 3, 4, and 5 weeks, respectively. Additionally, thermal withdrawal latency increased in the exercised group compared with the control group, with a MD of 2.48 (95% CI 0.59–4.38), 3.57 (95% CI 2.10–5.05), 3.92 (95% CI 2.82–5.03), and 2.84 (95% CI 1.29–4.39) at 1, 2, 3, and 4 weeks, respectively. Subgroup analyses were performed for pain models, exercise start point, exercise forms, and duration, which decreased heterogeneity to some extent.

**Conclusion:** This meta-analysis indicated that exercise provoked an increase in mechanical withdrawal threshold and thermal withdrawal latency in animal NP models. Exercise therapy may be a promising non-pharmacologic therapy to prevent the development of NP. Further, preclinical studies focused on improving experiment design and reporting are still needed.

## Introduction

Neuropathic pain (NP) is an important public health issue, with a prevalence ranging from 3.3 to 8.2% (1). According to a systematic review of epidemiological study, NP in a general population can reach 10% (2). In 2008, NP has been redefined as pain caused by a lesion or disease of the somatosensory system by the Assessment Committee of the NP Special Interest Group (NeuPSIG) (3). Various causes for NP have been observed, including trauma, infection (e.g., herpes zoster virus), metabolic abnormality (e.g., diabetic neuropathy), neurotoxin (e.g., chemotherapy drug), and tumor infiltration. Patients with NP exhibit a complex combination of symptoms, such as partial or complete loss of sensation, hyperpathia, and allodynia. Patients with conditions frequently experience daily pain that greatly reduces their health-related quality of life. The incidence of emotional disorders such as depression and anxiety also increases (4).

NP is effectively treated differently. Drugs, such as analgesics and antidepressants, are relatively ineffective in coping with NP, and long-term pharmacotherapy in patients always experience problematic adverse effects (5, 6). NP treatments require increased attention to multidisciplinary pain management, including some non-pharmacologic therapies (7). NP rehabilitation has focused on exercise therapy, but minimal agreement is found in literature. Exercise therapy applied in reducing NP is still relatively new, and the effects have not been confirmed in patients. In recent years, multiple animal experiments studies on the effects of exercise have been published. To the best of our knowledge, no systematic review and meta-analysis have been performed to explore the effects of exercise on NP. In the present study, we used NP rat models induced by peripheral nerve injury to determine the effects of exercise, summarize experimental designs, and provide a basis for clinical studies.

## Materials and Methods

This systematic review and meta-analysis complied with PRISMA (Preferred Reporting Items for Systematic Reviews and Meta-Analyses) guidelines (Supplementary Table 1) (8). We registered the protocol on PROSPERO, and the registration number is CRD42019124689. The record can be accessed at https://www.crd.york.ac.uk/PROSPERO.

### Search Strategy

PubMed, EMBASE, and Web of Science were searched from the earliest available date to January 2019. Key search terms included neuralgia, neurodynia, NP, nerve pain, sciatica, nerve crush, nerve ligation, nerve constriction, peripheral neuropathy, exercise, locomotion, run, swim, and environmental enrichment. The detailed search strategies are available in Supplementary Table 2. The reference lists of included studies were reviewed manually for other potentially relevant citations. No limits on language, publication date, or publication status were applied.

### Inclusion Criteria for Studies

#### Types of Studies

Controlled comparative animal studies investigating the effects of exercise in NP rat models induced by peripheral nerve injury were included.

#### Types of Animals

We included NP rat models induced by peripheral nerve injury, such as sciatic nerve ligation (SNL), chronic constriction injury (CCI), sciatic nerve injury, and diabetic neuropathy. Each model displayed at least one of the pathophysiological characteristics of NP. Considering the potential differences in the underlying mechanisms and treatments between adults and infants, infant nerve injury models were excluded.

#### Types of Interventions

The intervention groups used exercise therapy. The forms of exercise could be voluntary, for example, voluntary wheel running, and environmental enrichment and forced, for example, treadmill running, swimming, and forced wheel running. The exercised group referred to NP rats with exercise therapy, while the control group included NP rats without any exercise.

#### Types of Outcome Measurements

Pain-related behavioral test was reported as outcome measures, for example, mechanical allodynia, thermal hyperalgesia, and cold allodynia.

### Study Selection

Two researchers independently searched the database according to the search strategies and imported all the records into Endnote software. Evaluators included articles by removing duplications, screening titles and abstracts, and assessing full texts based on the inclusion criteria for studies. Disagreements were solved together by discussion.

### Data Extraction

Two pairs of researchers independently extracted and cross-checked data of all the included studies by using a standardized data extraction table. The following information were extracted: general information, such as the last name of the first author, and publication year, characteristics of the rats, such as rat species, gender, age, and weight, pain models, exercise program, such as exercise forms, start point, intensity, and duration, control intervention, and behavioral tests. Data on findings and proposed mechanisms and the risk of bias were also extracted. Pain behavior outcomes, including mean value, standard deviation, and the number of rats per group, were extracted for each study. If relevant data were unavailable in the articles, we would contact the corresponding author to obtain the original data. However, if a response was not available, we would use Getgata software to obtain the data showed by graphs. Disagreements were resolved by discussions.

### Assessment of Risk of Bias

The methodological quality of all included studies was evaluated using Systematic Review Center for Laboratory Animal Experimentation's (SYRCLE) risk of bias tool for animal studies (9). This tool is composed of 10 items and reflects the 6 aspects of the risk of bias: (1) selection bias (sequence generation, baseline characteristics, and allocation concealment), (2) performance bias (random housing, and blinding), (3) detection bias (random outcome assessment, and blinding), (4) attrition bias (incomplete outcome data), (5) reporting bias (selective outcome reporting), and (6) other sources of bias. Indicating “yes” means a low risk of bias, “no” means a high risk of bias, and “unclear” means no sufficient details to measure the risk of bias.

### Statistical Analysis

We imported the quantitative data into Review Manager 5.3. The outcome measure of pain-related behavioral test was a continuous outcome. Mean differences (MDs) with 95% confidence intervals (CIs) were calculated. We used random-effects models for pooled-effect estimates, which considers the variation between studies and weighs each study accordingly. Between-study heterogeneity was measured via Chi^2^ statistic (*P* < 0.1) and quantified with *I*^2^ statistic (10). I^2^ values <25, 50%, and over 50% indicate low, moderate, and high heterogeneity, respectively (10). Subgroup analyses were performed as predefined in the protocol aimed at reducing heterogeneity. Variables included pain models, exercise forms, start point, and duration. Moreover, sensitivity analysis was performed to assess the robustness of the findings, and we considered the effect of the hormone. We assessed the possibility of publication bias by using Egger's regression analysis (11). Values of *P* < 0.05 were considered statistically significant.

## Results

**Figure 1:**
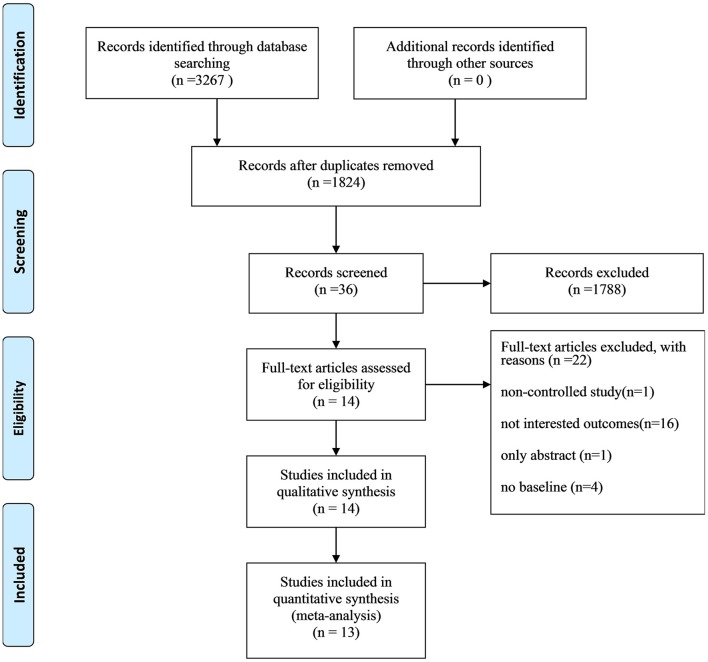
Flow chart of the study selection procedure.

A study identification and selection process flow chart is shown in Figure 1. We identified 3,267 records through database searching and other sources. After removing duplications, 1,824 records remained. The researchers then screened the records based on titles and abstracts, and the remaining 36 records were reviewed for full-text reading. As a result, 22 studies were excluded because they were non-controlled studies, their outcomes were not of our interest, only their abstracts were available, or their study involved no baseline. An additional 14 articles (12–25) were included, and 13 articles (12–20, 22–25) were pooled for meta-analysis.

### Study Characteristics

Table 1 summarizes the basic characteristics of the 14 eligible studies (12–25), all of which had been published between 2007 and 2018. All of the studies involved 268 rats, 137 and 131 of which were in the exercise (51.1%) and control groups (48.9%), respectively. The two species of rats in the included studies were Sprague–Dawley (78.6%) and Wistar rats (21.4%). Only one study (22) (7.1%) used female rats, and 13 studies (12–21, 23–25) (92.9%) used male rats in their experiments. The age of the rats was mentioned in 3 studies (17, 18, 22), which ranged from 6 weeks old to 12 weeks old, and 4 articles (13, 23–25) described as adult rats. A total of 12 studies (12–17, 19–21, 23–25) described the weight of rats, which ranged from 180 to 380 g. Three pain models were reported in the different studies, which included SNL (21.4%), CCI (50%), and diabetic neuropathy (28.6%). The included studies used mechanical withdrawal threshold and thermal withdrawal latency as outcome data.

**Table 1:** Characteristics of included studies.

**References**	**Species**	**Gender**	**Age(week)** **/weight(g)**	**Pain model**	**Exercise type**	**Start point**	**Exercise intensity**	**Exercise duration**	**Control intervention**	**Behavioral tests**
Kuphal et al. (12)	SD rats	Male	NR/250–300	SNL	Swimming	2 weeks before surgery	Increase from 10 min/session to 90 min/session, for 90 min/day	39 d	No exercise	TWL
Shankarappa et al. (13)	SD rats	Male	Adult/200	Diabetic neuropathy	Treadmill running	5 days before streptozotocin administration	18 m/min, with 0° slope, for 60 min/day, 5 day/week	10 weeks	No exercise	MWT
Stagg et al. (14)	SD rats	Male	NR/250–380	SNL	Treadmill running	The day of surgery	Increase from 14 m/min to 16 m/min with 8% grade, for 30 min/day, 5 day/week	5 weeks	No exercise	MWT, TWL
Chen et al. (15)	SD rats	Male	NR/250–300	CCI	Treadmill running or swimming	2 days before surgery	Treadmill running: increase from 20 m/min to 30 m/min, with 0°slope; from 15 min/d to 60 min/day, 5 day/week; swimming: increase from 10 min/session to 90 min/session, for 90 min/day	Treadmill running: 6 weeks; swimming: 39 day	No exercise	MWT, TWL
Chen et al. (16)	Wistar rats	Male	NR/285–335	Diabetic Neuropathy	Treadmill running	3 days before surgery	Increase from 20 m/min to 25 m/min, for 30 min/day to 60 min/day, 7 day/week	8 weeks	No exercise	MWT, TWL
Kim et al. (17)	SD rats	Male	10/250–300	CCI	Treadmill running	1 week before surgery	Increase from 8 m/min to 22 m/min, for 30 min/day, 5 day/week	4 weeks	No exercise	MWT, TWL
Grace et al. (18)	SD rats	Male	10–12/NR	CCI	Voluntary wheel running	the day of surgery	single-housed with in-cage running wheels	11 weeks	locked wheels	MWT
Hung et al. (19)	SD rats	Male	NR/220–270	CCI	Treadmill running	3 days after surgery	Increase from 14 to 16 m/min with an 8% grade, for 30 min/day, 5 day/week	4 weeks	No exercise	MWT, TWL
Huang et al. (20)	SD rats	Male	NR/220–270	CCI	Treadmill running	8 days after surgery	Increase from 14 to 16 m/min, with 8% grade, for 30 min/day, 7 day/week	3 weeks	No exercise	MWT, TWL
Tsai et al. (21)	SD rats	Male	NR/285–335	CCI	Treadmill running	6 days after surgery	Increase from 14 m/min to 16 m/min, with 8% grade, for 30 min/day, 7 day/week	3 weeks	No exercise; treadmill running with 0% grade	MWT, TWL
Yamaoka et al. (22)	Wistar rats	Female	6–8/NR	SNL	Treadmill running	2 day after surgery	Increase from 10 m/min to 20 m/min with 10° slope, for 10 min/day, 5 day/week	6 weeks	No exercise	MWT, TWL
Farzad et al. (23)	Wistar rats	Male	Adult/180–220	CCI	Swimming	1 week before surgery	Increase from 10 min/session to 30 min/session, for 10–60 min/day, 5 day/week	4 weeks	No exercise	MWT, TWL
Ma et al. (25)	SD rats	Male	Adult/200–250	Diabetic neuropathy	Treadmill running	The week of streptozotocin administration	Increase from 5 m/min to 10 m/min with 10% grade, 10 min/day, 4 day/week	5 weeks	No exercise	MWT
Ma et al. (24)	SD rats	Male	Adult/200–250	Diabetic neuropathy	Treadmill running	The week of streptozotocin administration	Increase from 5 m/min to 10 m/min, with 10% grade, 10 min/day, 4 day/week	5 weeks	No exercise	MWT

*SD, Sprague Dawley; NR, no record; CCI, chronic constriction injury; SNL, partial sciatic nerve ligation; TWL, Thermal withdrawal latency; MWT, Mechanical withdrawal threshold*.

Two types of exercise were employed in the 14 studies (12–25). One study (18) used voluntary exercise, and the remaining studies used forced exercise. Grace et al. (18) used voluntary wheel running as exercise form, and the rats in the experimental group were housed with running wheels, whereas control rats were housed with locked wheels. Treadmill running and swimming were the forced exercise training in 10 (13, 14, 16, 17, 19–22, 24, 25) and 2 studies (12, 23), respectively. Only one study (15) used both treadmill running and swimming for the research. Six studies (12, 13, 15–17, 23) showed a period of habituation to training between 2 days and 2 weeks. However, the rats were allowed to recover between 2 and 8 days after surgery in 6 studies (19–22, 24, 25) and then training commenced. Another 2 studies (14, 18) started training at surgery day or streptozotocin administration. The running protocols consisted of inclination, velocity, and duration. Rats in seven studies (14, 19–22, 24, 25) were forced to run with inclination, whereas 8 and 10% grades were used in the 4 (14, 19–21) and 3 studies (22, 24, 25), respectively. The velocity of running involved a gradual transition from 5 m/min to 30 m/min. The running duration was between 10 and 60 m/min, 4 and 7 day/week, and 3 and 10 weeks. Considering the swimming protocols, three studies (12, 15, 23) were included. Two studies (12, 15) underwent swimming training for 90 min/day for 39 days. The time duration in the study of Farzad et al. (23) increased from 10 min/day to 60 min/day for 4 weeks. Among the 14 studies, non-exercise was used as the control intervention in 13 studies (12–17, 19–25), and the remaining one study (18) adopted locked wheels as a control intervention.

### Study Quality Assessment

The results of risk of bias for each study is summarized in Table 2. The risk of bias on randomization and allocation concealment in all the studies were inadequate to judge. Baseline characteristics in the 14 studies were assessed as low risk. Baseline characteristics, such as initial weight, was described in the included studies, which suggested that no significant differences between the experiment and control groups were observed. Blinding of caregivers and researchers were incompletely described in all the studies. Five studies (15, 16, 18, 20, 21) (7%) provided information relating to blinding for outcome assessment. In addition, random housing, random outcome assessment, incomplete outcome data, and selective outcome reporting were assessed as unclear risk.

**Table 2:** SYRCLE's risk of bias tool for animal studies.

**References**	**Sequence generation**	**Baseline characteristics**	**Allocation concealment**	**Random housing**	**Performance Blinding**	**Random outcome assessment**	**Blinding of outcome assessment**	**Incomplete outcome data**	**Selective outcome reporting**	**Other sources of bias**
Kuphal et al. (12)	U	Y	U	U	N	U	U	U	U	Y
Shankarappa et al. (13)	U	Y	U	U	N	U	U	U	U	Y
Stagg et al. (14)	U	Y	U	U	N	U	U	U	U	Y
Chen et al. (15)	U	Y	U	U	N	U	Y	U	U	Y
Chen et al. (16)	U	Y	U	U	N	U	Y	U	U	Y
Kim et al. (17)	U	Y	U	U	N	U	U	U	U	Y
Grace et al. (18)	U	Y	U	U	N	U	Y	U	U	Y
Hung et al. (19)	U	Y	U	U	N	U	U	U	U	Y
Huang et al. (20)	U	Y	U	U	N	U	Y	U	U	Y
Tsai et al. (21)	U	Y	U	U	N	U	Y	U	U	Y
Yamaoka et al. (22)	U	Y	U	U	N	U	U	U	U	Y
Farzad et al. (23)	U	Y	U	U	N	U	U	U	U	Y
Ma et al. (25)	U	Y	U	U	N	U	U	U	U	Y
Ma et al. (24)	U	Y	U	U	N	U	U	U	U	Y

*Y, yes = low risk of bias; N, no = high risk of bias; U, unclear = unclear risk of bias*.

### Meta-Analysis of the Efficacy of Treatment With Exercise

#### Mechanical Withdrawal Threshold

**Figure 2:**
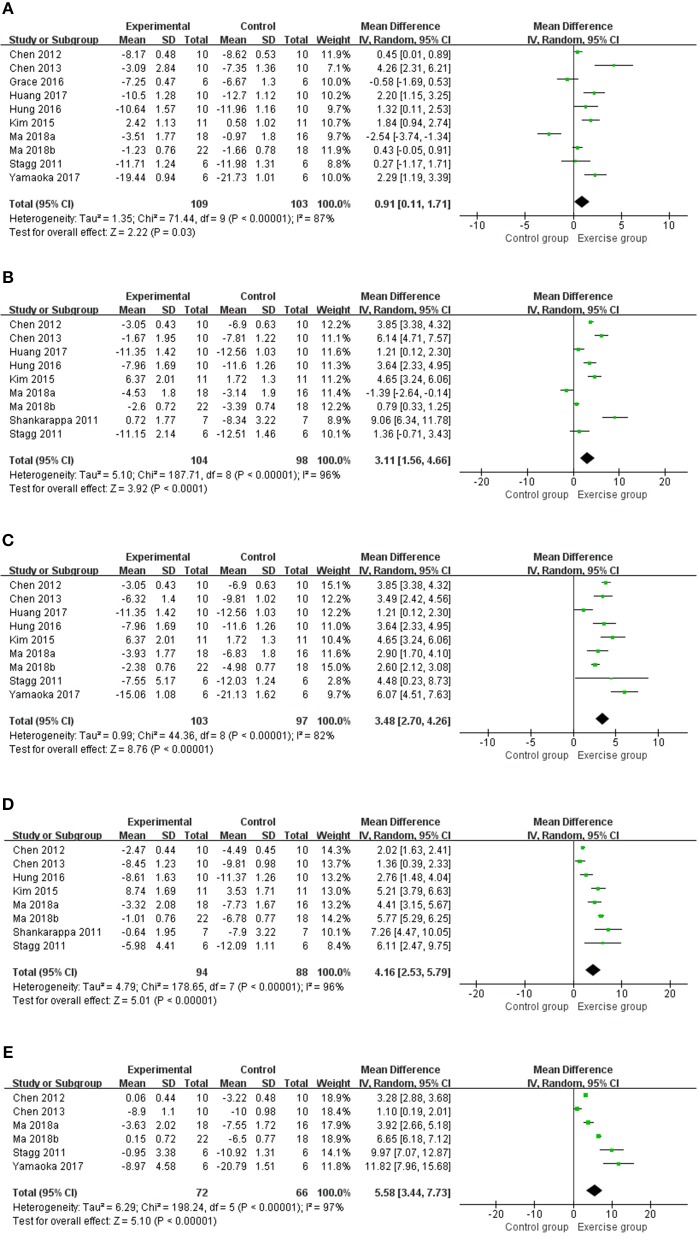
Meta-analysis estimate for exercise on mechanical withdrawal threshold. **(A)** Effect estimates were reported at 1 week; **(B)** effect estimates were reported at 2 weeks; **(C)** effect estimates were reported at 3 weeks; **(D)** effect estimates were reported at 4 weeks; **(E)** effect estimates were reported at 5 weeks. CI, confidence interval; IV, inverse variance. Data are reported as pooled mean differences by using the inverse variance method with random-effects models.

A total of 11 studies (13–20, 22, 24, 25) with 226 rats investigating the effect of exercise on mechanical withdrawal threshold in pain models were involved in the meta-analysis. Overall, the results showed exercise increased mechanical withdrawal threshold at 1 week (*n* = 212, *MD* = 0.91, 95% CI 0.11–1.71, I^2^ = 87%), 2 weeks (*n* = 202, *MD* = 3.11, 95% CI 1.56–4.66, I^2^ = 96%), 3 weeks (*n* = 200, *MD* = 3.48, 95% CI 2.70–4.26, I^2^ = 82%), 4 weeks (*n* = 182, *MD* = 4.16, 95% CI 2.53–5.79, I^2^ = 96%), and 5 weeks (*n* = 138, *MD* = 5.58, 95% CI 3.44–7.73, I^2^ = 97%) (Figure 2) The results of subgroup analyses are described in Table 3 and revealed that the effects varied across different NP models and exercise programs (start point, and duration). For example, subgroup analysis for NP models found that exercise can increase mechanical withdrawal thresholds in CCI, SNL, and DPN models after 2 weeks. Additionally, subgroup analysis for the exercise start point, we found that the effect was related to the presence or absence of habituation to exercise. The training group before modeling reported positive results at 1 week (*n* = 62, *MD* = 1.93, 95% CI 0.24–3.63, I^2^ = 90%) and 2 weeks (*n* = 72, *MD* = 5.57, 95% CI 3.83–7.31, I^2^ = 86%), but no remarkable difference was found in the group of after modeling at 1 week (*n* = 126, *MD* = 0.75, 95% CI −0.68 to 2.18, I^2^ = 91%) and 2 weeks (*n* = 114, *MD* = 1.04, 95% CI −0.49 to 2.58, I^2^ = 90%). We found that the effect was independent from the exercise duration by subgroup analysis according to exercise duration. At 1 week, the results revealed that the effect was significant (*n* = 62, *MD* = 1.83, 95% CI 1.23–2.42, I^2^ = 0%) in <4 weeks duration, but did not appear to be effective (*n* = 150, *MD* = 0.52, 95% CI −0.45 to 1.49, I^2^ = 89%) in more than 5 weeks duration.

**Table 3:** Summary of results.

**Time point**	**Subgroup**		**No of trials**	**No of rats**	**Mean difference (95% CI)**	**Heterogeneity *I^**2**^*(%)**	**Effect size** ***P***
**MECHANICAL WITHDRAWAL THRESHOLD**							
1 week	Modeling	CCI	5	94	1.03 [0.13, 1.93]	81	0.03
		SNL	2	24	1.34 [−0.64, 3.31]	79	0.19
		DPN	3	94	0.60 [−2.19, 3.40]	95	0.67
	Start point	Before modeling	3	62	1.93 [0.24, 3.63]	90	0.03
		After modeling	5	126	0.75 [−0.68, 2.18]	91	0.31
		The day of modeling	2	24	−0.27 [−1.14, 0.61]	0	0.55
	Duration	≤4 weeks	3	62	1.83 [1.23, 2.42]	0	<0.00001
		≥5 weeks	7	150	0.52 [−0.45, 1.49]	89	0.30
	Overall		10	212	0.91 [0.11, 1.71]	87	0.03
2 weeks	Modeling	CCI	4	82	3.32 [1.97, 4.67]	86	<0.00001
		DPN	4	108	3.46 [0.05, 6.87]	97	0.05
	Start point	Before modeling	4	72	5.57 [3.83, 7.31]	86	<0.00001
		After modeling	4	114	1.04 [−0.49, 2.58]	90	0.18
	Duration	≤4 weeks	3	62	3.13 [1.04, 5.22]	88	0.003
		≥5 weeks	6	140	3.12 [1.01, 5.24]	97	0.004
	Overall		9	202	3.11 [1.56, 4.66]	96	<0.0001
3 weeks	Modeling	CCI	4	82	3.32 [1.97, 4.67]	86	<0.00001
		SNL	2	24	5.88 [4.42, 7.34]	0	<0.00001
		DPN	3	94	2.80 [2.33, 3.28]	12	<0.00001
	Start point	Before modeling	3	62	3.86 [3.45, 4.28]	0	<0.00001
		After modeling	5	126	3.18 [1.97, 4.39]	85	<0.00001
	Duration	≤4 weeks	3	62	3.13 [1.04, 5.22]	88	0.003
		≥5 weeks	6	138	3.66 [2.79, 4.53]	81	<0.00001
	Overall		9	200	3.48 [2.70, 4.26]	82	<0.00001
4 weeks	Modeling	CCI	3	62	3.24 [1.45, 5.02]	89	0.0004
		DPN	4	108	4.56 [2.07, 7.04]	96	0.0003
	Start point	Before modeling	4	76	3.56 [1.76, 5.36]	91	0.0001
		After modeling	3	94	4.38 [2.54, 6.23]	90	<0.00001
	Duration	≤4 weeks	2	42	3.96 [1.56, 6.36]	84	0.001
		≥5 weeks	6	140	4.25 [2.24, 6.27]	97	<0.0001
	Overall		8	182	4.16 [2.53, 5.79]	96	<0.00001
5 weeks	Modeling	SNL	2	24	10.64 [8.32, 12.96]	0	<0.00001
		DPN	3	94	3.90 [0.18, 7.62]	98	0.04
	Start point	Before modeling	2	40	2.23 [0.10, 4.36]	95	0.04
		After modeling	3	86	6.77 [4.09, 9.45]	92	<0.00001
	Overall		6	138	5.58 [3.44, 7.73]	97	<0.00001
**THERMAL WITHDRAWAL LATENCY**							
1 week	Modeling	CCI	5	96	3.00 [0.52, 5.48]	94	0.02
		SNL	2	24	1.82 [−0.23, 3.87]	0	0.08
	Exercise type	Running	5	94	2.63 [−0.56, 5.82]	95	0.009
		Swimming	3	46	1.98 [0.37, 3.60]	69	0.11
	Start point	Before modeling	5	88	1.67 [0.29, 3.05]	77	0.02
		After modeling	2	40	4.88 [0.04, 9.72]	95	0.05
	Duration	≤4 weeks	4	76	2.89 [−0.84, 6.62]	96	0.13
		≥5 weeks	4	64	2.02 [0.43, 3.61]	76	0.01
	Overall		8	140	2.48 [0.59, 4.38]	93	0.01
2 weeks	Modeling	CCI	5	96	4.46 [2.60, 6.32]	89	<0.00001
		SNL	2	24	1.53 [−1.55, 4.61]	41	0.33
	Exercise type	Running	5	94	3.96 [1.43, 6.49]	92	0.002
		Swimming	3	46	2.86 [2.18, 3.55]	0	<0.00001
	Start point	Before modeling	5	88	2.54 [1.97, 3.11]	0	<0.00001
		After modeling	2	40	6.85 [4.09, 9.62]	82	<0.00001
	Duration	≤4 weeks	4	76	4.93 [2.54, 7.32]	89	<0.0001
		≥5 weeks	4	64	2.32 [1.47, 3.16]	23	<0.00001
	Overall		8	140	3.57 [2.10, 5.05]	87	<0.00001
3 weeks	Modeling	CCI	5	96	4.33 [3.38, 5.28]	40	<0.00001
		SNL	2	24	4.25 [2.09, 6.42]	0	0.0001
	Exercise type	Running	5	94	3.94 [2.07, 5.81]	81	<0.0001
		Swimming	3	46	3.60 [2.32, 4.89]	44	<0.00001
	Start point	Before modeling	5	88	3.35 [2.14, 4.56]	63	<0.00001
		After modeling	2	40	5.22 [3.89, 6.54]	19	<0.00001
	Duration	≤4 weeks	4	76	4.79 [3.88, 5.69]	0	<0.00001
		≥5 weeks	4	64	2.96 [1.63, 4.29]	60	<0.0001
	Overall		8	140	3.92 [2.82, 5.03]	69	<0.00001
4 weeks	Modeling	CCI	4	76	3.36 [0.94, 5.77]	92	0.006
		SNL	2	24	2.32 [−1.43, 6.07]	69	0.23
	Exercise type	Running	4	74	3.66 [1.55, 5.76]	76	0.0007
		Swimming	3	46	2.45 [0.54, 4.36]	84	0.01
	Start point	Before modeling	5	88	2.08 [0.43, 3.73]	86	0.01
	Duration	≤4 weeks	3	56	4.39 [3.23, 5.54]	30	<0.00001
		≥5 weeks	4	64	1.48 [0.24, 2.71]	56	0.02
	Overall		7	120	2.84 [1.29, 4.39]	86	0.0003

*CCI, chronic constriction injury; SNL, partial sciatic nerve ligation; DPN, diabetic peripheral neuropathy*.

#### Thermal Withdrawal Latency

**Figure 3:**
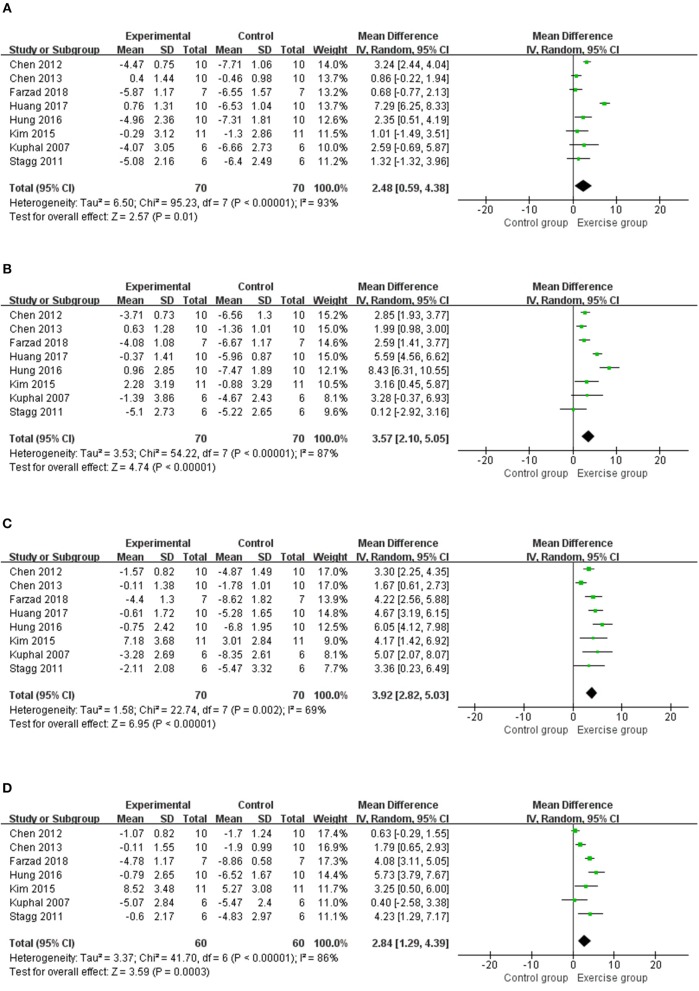
Meta-analytic estimate for exercise on thermal withdrawal latency. **(A)** Effect estimates were reported at 1 week; **(B)** effect estimates were reported at 2 weeks; **(C)** effect estimates were reported at 3 weeks; **(D)** effect estimates were reported at 4 weeks. CI, confidence interval; IV, inverse variance. Data are reported as pooled mean differences by using the inverse variance method with random-effects models.

Eight studies (12, 14–17, 19, 20, 23) with 140 rats were included in the meta-analysis (Figure 3), and the results found that the thermal withdrawal latency was increased in the exercised group compared with the control groups at 1 week (*n* = 140, *MD* = 2.48, 95% CI 0.59–4.38, I^2^ = 93%), 2 weeks (*n* = 140, *MD* = 3.57, 95% CI 2.10–5.05, I^2^ = 87%), 3 weeks (*n* = 140, *MD* = 3.92, 95% CI 2.82–5.03, I^2^ = 69%), and 4 weeks (*n* = 120, *MD* = 2.84, 95% CI 1.29–4.39, I^2^ = 86%). Subgroup analyses on different NP models and exercise programs, such as forms, start point, and duration, are shown in Table 3. For NP models, thermal withdrawal latencies were all significantly increased by exercise at 1 week (*n* = 96, *MD* = 3.00, 95% CI 0.52–5.48, I2 = 94%), 2 weeks (*n* = 96, *MD* = 4.46, 95% CI 2.60–6.32 I2 = 89%), 3 weeks (*n* = 96, *MD* = 4.33, 95% CI 3.38–5.28, *I*^2^ = 40%) and 4 weeks (*n* = 76, *MD* = 3.36, 95% CI 0.94–5.77, *I*^2^ = 92%) in CCI model, while only increased at 3 weeks (*n* = 24, *MD* = 4.25, 95% CI 2.09–6.42, *I*^2^ = 0%) in SNL model. The results of subgroup analysis on exercise duration in thermal withdrawal latency supported the conclusion of mechanical withdrawal threshold ( ≤ 4 weeks: *n* = 76, *MD* = 2.89, 95% CI −0.84 to 6.62, I^2^ = 96%; ≥5 weeks: *n* = 64, *MD* = 2.02, 95% CI 0.43–3.61, I^2^ = 76%).

### Sensitivity Analysis

To confirm the robustness of our results, we performed a sensitivity analysis. Estrogen can influence pain behaviors in animals. Mechanical pain thresholds of male and female rats change in estrogen-dependent manners (26). We excluded the study Yamaoka et al. (22) that used female rats. The results revealed an inverse at 1 week for assessing mechanical withdrawal threshold (*MD* = 0.75, 95% CI −0.08 to 1.58, *p* = *0.08*), but did not statistically alter the results at 3 and 5 weeks.

### Publication Bias Test

In the meta-analysis for mechanical withdrawal threshold, no publication bias was found at 1 week (*P* = 0.204, Egger test). However, subsequent publication bias was not assessed because <10 studies were included, which potentially interfered with the power of Egger test.

### Signaling Pathways

Among the 14 included studies, 13 studies (13–25) described the possible mechanisms of exercise in attenuating NP. The main signaling pathways were summarized in four aspects, namely, inflammatory response, ion channel, endogenous opioid system, and immune response, as shown in Table 4.

**Table 4:** Proposed mechanisms of included studies.

**References**	**Findings and proposed mechanisms**
Kuphal et al. (12)	NR
Shankarappa et al. (13)	Exercise reduced high-voltage activated Ca^2+^ current density
Stagg et al. (14)	Exercise increased β-endorphin, met-enkephalin
Chen et al. (15)	Exercise increased Hsp72; Exercise reduced TNF-α, IL-1β
Chen et al. (16)	Exercise increased Hsp72
Kim et al. (17)	Exercise reduced μ-opioid receptor
Grace et al. (18)	Exercise increased NLRP3, p65, P2X4R, p-p38, BDNF
Hung et al. (19)	Exercise increased IL-10; Exercise reduced IL-6, Iba1
Huang et al. (20)	Exercise increased IL-10; Exercise reduced TNF-α, IL-6
Tsai et al. (21)	Exercise increased IL-10; Exercise reduced IL-6, TNF-α
Yamaoka et al. (22)	Exercise reduced Rnf34, Pacap
Farzad et al. (23)	Exercise increased GAD65; Exercise reduced Irisin
Ma et al. (25)	Exercise reduced IL-1β, IL-6, TNF-α, IL-1R, IL-6R, TNFR1
Ma et al. (24)	Exercise reduced mTOR, IL-6, S6K1, 4E-BP1

*NR, no record; Hsp, Heat shock protein; GAD, glutamate decarboxylase; TNF-α, tumor necrosis factor-α; IL, interleukin; p-p38, Phosphorylated p38; BDNF, Brain derived neurotrophic factor; Iba1, ionized calcium binding adaptor molecule 1; IL-1R, interleukin-1 receptor; TNFR1, tumor necrosis factor receptor 1; mTOR, mammalian target of rapamycin*.

## Discussion

This study is the first to systematically review the effects of exercise on NP induced by peripheral nerve injury in rat models. Our finding was based on a thorough analysis of all the included studies. We proved that exercise training can increase mechanical withdrawal threshold and thermal withdrawal latency in NP models. Summary of results were seen in Table 3, a relation between the effect and the presence or absence of habituation to exercise has been demonstrated by subgroup analyses. However, total duration of exercise was independent. These results suggested that people who exercise may recover better from peripheral nerve injury than those who do not. The evidence was also supported by longitudinal population-based studies (27, 28). The prevalence of chronic pain is lower in those who reporting a history of regular exercise. Maintain exercising may make people more likely to have high pain thresholds. Regarding different forms of exercise, we planned to discuss the differences between forced exercise and voluntary exercise. However, only one included study (18) related to voluntary wheel running was performed. Thus, no comparison was made. Voluntary exercise means unrestricted access to running, and the paradigm in Grace et al. (18) has been demonstrated that it weakens the proinflammatory response to endotoxin and stress. Moreover, they found that voluntary wheel running after CCI progressively reduced NP. We then compared different forms of forced exercise, such as running and swimming, but no evidence has been demonstrated as to which one is more effective. In addition, different NP models may have different responses to exercise. The results in Table 3 showed that exercise can increase mechanical withdrawal thresholds in CCI, SNL and DPN models after 2 weeks, while thermal withdrawal latencies at 1 week, 2 weeks, 3 weeks, and 4 weeks were all significantly increased only in CCI model. These promising results supported exercise in managing NP induced by peripheral nerve injury in rat models.

Concerning the physiological explanations for exercise-induced benefits described above, 13 studies (13–25) have discussed the effects of exercise on the mechanisms of NP induced by peripheral nerve injury (Table 4). For example, six studies (15, 19–21, 24, 25) have shown that exercise decreased proinflammatory cytokine expressions, such as tumor necrosis factor-α (TNF-α), interleukin (IL)-1β, and IL-6, and increased the expression of anti-inflammatory cytokines, such as IL-10. However, exactly how exercise affected these cytokines has yet to be fully explored in these studies. Chen et al. (15) found that rats in CCI group shows lower Heat shock protein (Hsp) 72 and greater proinflammatory cytokines (TNF-α and IL-1β) in sciatic nerve than in normal group, but exercise reversed the expression of Hsp72, TNF-α, and IL-1β. Chen et al. (16) also found in diabetic neuropathy rats that increased Hsp72 alleviates pain. Experiments have confirmed that increased Hsp72 protects cells from different stresses and increases cellular survivability (29, 30). Some evidence has been found that Hsp72 can modulate proinflammatory cytokines and reduce their secretion through inactivating the pathway of nuclear factor kappa B (31).

This systematic review showed several limitations. First, substantial heterogeneity was observed among our included studies in the meta-analysis. Further subgroup analyses were conducted to reveal some possibilities of heterogeneity, such as models of NP and exercise forms and duration. Heterogeneity was in reduced varying degrees after subgroup analyses. Second, some methodological weaknesses in the included animal studies were observed, such as experimental designs, performance, and reporting methods, which could affect the authenticity and reliability of the results. We used SYRCLE's risk of bias tool to assess the quality of these animal studies and found the risk of bias could not be estimated in most of the studies, which indicated a high risk of bias. Egger test for assessing publication bias was performed, and the results of mechanical withdrawal threshold in 1 week indicated no potential publication bias.

Based on the results in this systematic review, we found that exercise could alleviate pain induced by peripheral nerve injury in CCI, SNL, and DPN rat models. Although limited by poor reported risk of bias and high level of heterogeneity, the current meta-analysis still highlighted that exercise might play an active role in NP induced by peripheral nerve injury. Exercise therapy may be a promising non-pharmacologic therapy to prevent the development of NP. Using the ARRIVE guidelines (32) and Gold Standard Publication Checklist (33) is also essential to improve the reporting and methodological quality of animal studies. Further preclinical studies focused on improving experiment design and reporting are still needed.

## Data Availability

The raw data supporting the conclusions of this manuscript will be made available by the authors, without undue reservation, to any qualified researcher.

## Author Contributions

PC and XW: conceptualization and supervision. YW and GS: methodology. JG and BC: validation and writing—original draft preparation. JG and YZhe: formal analysis. YW, ZY, GS, and YZhu: investigation. JG, YZhu, PC, and XW: writing—review and editing. BC and ZY: visualization.

### Conflict of Interest Statement

The authors declare that the research was conducted in the absence of any commercial or financial relationships that could be construed as a potential conflict of interest.

## Supplementary Material

Supplementary Table 1 PRISMA Checklist of this meta-analysis.

Supplementary Table 2 Search strategies for all databases.
