# Supplementary material for: Meta-Analysis of the Effect of Exercise on Neuropathic Pain Induced by Peripheral Nerve Injury in Rat Models
Source: Front Neurol. 2019 Jun 14;10:636. doi: 10.3389/fneur.2019.00636 (PMC6587136; doi:10.3389/fneur.2019.00636)
Supplement: Supplementary Table 2 — Search strategies for all databases. [file Table_2.DOCX]

**Supplementary Table 1. Search strategies for all databases**

| **1. Search Strategy for PubMed** |
| --- |
| #1. Neuralgia[Mesh] OR Neuralgia$[Title/Abstract] OR Neurodynia$[Title/Abstract] OR "Neuropathic pain$"[Title/Abstract] OR “Nerve pain$”[Title/Abstract] OR sciatica[Title/Abstract] |
| #2. "nerve crush"[Title/Abstract] OR "nerve cut"[Title/Abstract] OR "nerve constriction"[Title/Abstract] OR "nerve inflammation"[Title/Abstract] OR "nerve injury"[Title/Abstract] OR "nerve ligation"[Title/Abstract] OR "peripheral neuropathy"[Title/Abstract] OR "chronic constriction injury"[Title/Abstract] OR "diabetic neuropathy"[Title/Abstract] OR pre-diabetes[Title/Abstract] OR "metabolic syndrome"[Title/Abstract] OR "high-fat diet"[Title/Abstract] OR diet-induced[Title/Abstract] OR [chemotherapy-induced[Title/Abstract]](https://www-ncbi-nlm-nih-gov-nus.vtrus.net/pubmed/30470691) OR "paclitaxel-induced"[Title/Abstract] OR streptozotocin-induced[Title/Abstract] |
| #3. pain$[Title/Abstract] |
| #4. #2 AND #3 |
| #5. #1 OR #4 |
| #6. Exercise[Mesh] OR "Exercise therapy"[Mesh] OR Locomotion[Mesh] OR exercise$[Title/Abstract] OR locomotion[Title/Abstract] OR run*[Title/Abstract] OR swim*[Title/Abstract] OR "environmental enrichment"[Title/Abstract] OR treadmill[Title/Abstract] OR vibration[Title/Abstract] OR aerobic$[Title/Abstract] OR strength$[Title/Abstract] OR isometric$[Title/Abstract] OR isotonic$[Title/Abstract] OR isokinetic$[Title/Abstract] OR endurance[Title/Abstract] OR weight$[Title/Abstract] OR physiotherapy[Title/Abstract] OR resistance[Title/Abstract] OR train*[Title/Abstract] |
| #7 Species: [Other Animals](https://www-ncbi-nlm-nih-gov-nus.vtrus.net/pubmed) |
| #8. #5 AND #6 AND #7 |
| **2. Search Strategy for EMBASE** |
| #1. Neuralgia/exp OR Neuralgia$:ab,ti OR Neurodynia$:ab,ti OR "Neuropathic pain$":ab,ti OR "Nerve pain$":ab,ti OR sciatica:ab,ti |
| #2. "nerve crush":ab,ti OR "nerve cut":ab,ti OR "nerve constriction":ab,ti OR "nerve inflammation":ab,ti OR "nerve injury":ab,ti OR "nerve ligation":ab,ti OR "peripheral neuropathy":ab,ti OR "chronic constriction injury":ab,ti OR "diabetic neuropathy":ab,ti OR pre-diabetes:ab,ti OR "metabolic syndrome":ab,ti OR "high-fat diet":ab,ti OR diet-induced:ab,ti OR chemotherapy-induced:ab,ti OR "paclitaxel-induced":ab,ti OR streptozotocin-induced:ab,ti |
| #3. pain$:ab,ti |
| #4. #2 AND #3 |
| #5. #1 OR #4 |
| #6. Exercise/exp OR 'Exercise therapy'/exp OR Locomotion/exp OR exercise$:ab,ti OR locomotion:ab,ti OR run*:ab,ti OR swim*:ab,ti OR "environmental enrichment":ab,ti OR treadmill:ab,ti OR vibration:ab,ti OR aerobic$:ab,ti OR strength$:ab,ti OR isometric$:ab,ti OR isotonic$:ab,ti OR isokinetic$:ab,ti OR endurance:ab,ti OR weight$:ab,ti OR physiotherapy:ab,ti OR resistance:ab,ti OR train*:ab,ti  #7. 'animal model'/de |
| #8. #5 AND #6 AND #7 |
| **3. Search Strategy for Web of Science** |
| #1 TS=( Neuralgia$ OR Neurodynia$ OR "Neuropathic pain$" OR sciatica) |
| #2 TS=("nerve crush" OR "nerve cut" OR "nerve constriction" OR "nerve inflammation" OR "nerve injury" OR "nerve ligation" OR "peripheral neuropathy" OR "chronic constriction injury" OR "diabetic neuropathy" OR pre-diabetes OR "metabolic syndrome" OR "high-fat diet" OR diet-induced OR chemotherapy-induced OR "paclitaxel-induced" OR streptozotocin-induced) |
| #3. TS=(pain$) |
| #4. #2 AND #3 |
| #5. #1 OR #4 |
| #6. TS=(exercise$ OR locomotion OR run* OR swim* OR "environmental enrichment" OR treadmill OR vibration OR aerobic$ OR strength$ OR isometric$ OR isotonic$ OR isokinetic$ OR endurance OR weight$ OR physiotherapy OR resistance OR train*) |
| #7. ALL=(animal$ OR rat$ OR rodent$) |
| #8. #5 AND #6 AND #7 |
| Timespan=All years. Databases=SCI-EXPANDED. Article Type= Article |
